# Comparative analysis of self-stimulatory behaviors in ASD and ADHD

**DOI:** 10.1192/j.eurpsy.2024.471

**Published:** 2024-08-27

**Authors:** B. A. Oroian, G. Costandache, E. Popescu, P. Nechita, A. Szalontay

**Affiliations:** ^1^Institute of Psychiatry “Socola” Iasi, Iasi, Romania

## Abstract

**Introduction:**

The phenomenon of self-stimulatory behaviors, commonly referred to as “stimming,” presents a fascinating avenue of exploration within the context of neurodevelopmental disorders. While stimming behaviors are widely associated with ASD, there is emerging evidence suggesting that individuals with ADHD may also engage in similar behaviors. This study seeks to undertake a comprehensive investigation of the neurophenomenology of stimming in individuals diagnosed with ASD and ADHD, aiming to discern potential shared and distinctive characteristics.

**Objectives:**

The principal objective of this research is to conduct an intricate neurophenomenological analysis of stimming behaviors in cohorts diagnosed with ASD (n=60) and ADHD (n=60), with a concurrent control group of neurotypical individuals (n=60). The study aspires to delineate the prevalence, typology, and neurophysiological underpinnings of stimming behaviors in both ASD and ADHD populations. Moreover, this study endeavors to identify whether particular stimming behaviors exhibit differential prevalence or intensity between the two disorders.

**Methods:**

Participants underwent rigorous neurophenomenological assessments, incorporating structured interviews, validated self-report questionnaires and direct observations. Diagnostic confirmation was established through the administration of gold-standard instruments, such as the Autism Diagnostic Observation Schedule (ADOS-2) for ASD and the Conners’ Parent Rating Scale for ADHD. Stimming behaviors were meticulously categorized (e.g., motor, vocal, sensory) and scrutinized for quantitative metrics, including frequency, duration, and complexity.

**Results:**

Preliminary analyses have uncovered profound disparities in the manifestation of stimming behaviors between ASD and ADHD cohorts. Individuals with ASD displayed a significantly higher prevalence of stimming behaviors, with motor stimming predominating, followed by vocal and sensory manifestations. In contrast, individuals with ADHD exhibited a comparatively reduced frequency and intensity of stimming, primarily within the motor domain, albeit notably less elaborate. Control group participants exhibited a negligible occurrence of stimming behaviors.

**Conclusions:**

This multidimensional exploration illuminates the nuanced neurophenomenological distinctions in self-stimulatory behaviors between ASD and ADHD. Stimming emerges as a pivotal feature in ASD, while its presence in ADHD, though discernible, is markedly attenuated. This study’s findings hold implications for precise diagnostic delineation and the prospect of personalized interventions for these complex neurodevelopmental conditions. Future avenues of research may delve into the neural substrates underpinning stimming behaviors, further enhancing our comprehension of these phenomena.

**Disclosure of Interest:**

None Declared

